# Ph染色体阴性骨髓增殖性肿瘤加速/急变期患者的临床特征及预后因素分析

**DOI:** 10.3760/cma.j.issn.0253-2727.2023.04.003

**Published:** 2023-04

**Authors:** 欣 严, 铁军 秦, 冰 李, 士强 曲, 丽娟 潘, 富慧 李, 宁宁 柳, 志坚 肖, 泽锋 徐

**Affiliations:** 中国医学科学院血液病医院（中国医学科学院血液学研究所），实验血液学国家重点实验室，国家血液系统疾病临床医学研究中心，天津医学健康研究院，细胞生态海河实验室，天津 300020 State Key Laboratory of Experimental Hematology, National Clinical Research Centre for Blood Diseases, Haihe Laboratory of Cell Ecosystem, Tianjin Institutes of Health Science, Institute of Hematology and Blood Diseases Hospital, Chinese Academy of Medical Science & Peking Union Medical College, Tianjin 300020, China

**Keywords:** 骨髓增殖性肿瘤, 加速期, 急变期, 临床特征, 预后因素, Myeloproliferative neoplasm, Accelerated phase, Blast phase, Clinical characteristics, Prognostic factors

## Abstract

**目的:**

分析Ph染色体阴性骨髓增殖性肿瘤加速期/急变期（MPN-AP/BP）患者的临床特征及预后因素。

**方法:**

收集2014年2月至2021年12月期间就诊于中国医学科学院血液病医院的67例Ph染色体阴性MPN-AP/BP患者的病例资料，回顾性分析其临床特征及预后因素。

**结果:**

①全部67例MPN-AP/BP患者中男31例（46.3％），女36例（53.7％），中位年龄为60（33～75）岁；原发性骨髓纤维化（PMF）进展的患者48例（PMF-AP/BP组），由真性红细胞增多症（PV）、原发性血小板增多症（ET）、骨髓增殖性肿瘤不能分类（MPN-U）进展的患者19例（其他MPN-AP/BP组）。PMF-AP/BP组与其他MPN-AP/BP组比较，乳酸脱氢酶（LDH）水平较高（925.9 U/L对576.2 U/L，*P*＝0.011)，脾肿大患者占比较高（81.4％对57.9％，*P*＝0.05），骨髓网状纤维≥2级者占比较高（93.6％对63.2％，*P*＝0.004），进展为AP/BP时间较短（28.7个月对81.0个月，*P*＝0.001）。②67例Ph染色体阴性MPN-AP/BP患者中，41例（61.2％）检出JAK2V617F突变，13例（19.4％）检出CALR外显子9突变（1型CALR突变11例，2型CALR突变2例），3例（4.5％）检出MPLW515突变，JAK2、MPL和CALR基因突变均未检出10例（14.9％）；非驱动基因突变检出率较高的依次为ASXL1（42.2％，27例）、SRSF2（25.0％，16例）、SETBP1（22.6％，15例）、TET2（20.3％，13例）、RUNX1（20.3％，13例）和TP53基因（17.2％，11例）。PMF-AP/BP组ASXL1基因突变检出率（51.1％对21.1％，*P*＝0.03）及SRSF2基因突变频率（VAF）高于其他MPN-AP/BP组（48.8％对39.6％，*P*＝0.008）。③多因素分析示复杂染色体核型是影响MPN-AP/BP患者总生存（OS）期的独立不良预后因素（*HR*＝2.53，95％*CI* 1.06～6.05，*P*＝0.036）。接受异基因造血干细胞移植或白血病样化疗的患者与接受支持治疗的患者比较，OS期较长［（21.3（95％*CI* 10.2～32.3）个月对3.0（95％*CI* 2.3～3.7）个月，*P*＝0.05；13.0（95％*CI* 8.3～17.7）个月对3.0（95％*CI* 2.3～3.7）个月，*P*＝0.011］。

**结论:**

与其他Ph染色体阴性MPN-AP/BP患者比较，PMF-AP/BP患者具有较高的脾肿大和骨髓网状纤维≥2级发生率、较高的LDH水平，进展为AP/BP时间也较短。复杂染色体核型是影响MPN-AP/BP患者OS期的独立不良预后因素。异基因造血干细胞移植和白血病样化疗可延长MPN-AP/BP患者OS期。

## Abstract (en)

**Objective:**

To evaluate the clinical characteristics and prognostic factors of patients with Philadelphia-negative myeloproliferative neoplasm-accelerated phase/blast phase（MPN-AP/BP）.

**Methods:**

A total of 67 patients with MPN-AP/BP were enrolled from February 2014 to December 2021 at the Institute of Hematology and Blood Diseases Hospital, Chinese Academy of Medical Sciences. Their clinical features and prognostic factors were analyzed retrospectively.

**Results:**

① Sixty-seven patients with MPN-AP/BP with a median age of 60（range, 33–75）years, including 31 males（46.3％）and 36 females（53.7％）, were analyzed. Forty-eight patients progressed from primary myelofibrosis（PMF）, and 19 progressed from other myeloproliferative neoplasms（MPNs）, which included polycythemia vera, essential thrombocythemia, and MPN unclassifiable. Patients who progressed from PMF had higher lactate dehydrogenase（LDH）levels than those who progressed from other MPNs（925.95 *vs*. 576.2 U/L, *P*＝0.011）, and there were higher proportions of patients who progressed from PMF with splenomegaly（81.4％ *vs*. 57.9％, *P*＝0.05）, a myelofibrosis grade of ≥2（93.6％ *vs*. 63.2％, *P*＝0.004）, and a shorter duration from diagnosis to the transformation to AP/BP（28.7 *vs*. 81 months, *P*＝0.001）. ② JAK2V617F, CALR, and MPLW515 were detected in 41（61.2％）, 13（19.4％）, and 3（4.5％）patients, respectively, whereas 10（14.9％）patients did not have any driver mutations（triple-negative）. Other than driver mutations, the most frequently mutated genes were ASXL1（42.2％, *n*＝27）, SRSF2（25％, *n*＝16）, SETBP1（22.6％, *n*＝15）, TET2（20.3％, *n*＝13）, RUNX1（20.3％, *n*＝13）, and TP53（17.2％, *n*＝11）. The ASXL1 mutation was more enriched（51.1％ *vs*. 21.1％, *P*＝0.03）, and the median variant allele fraction（VAF）of the SRSF2 mutation（median VAF, 48.8％ *vs*. 39.6％; *P*＝0.008）was higher in patients who progressed from PMF than those who progressed from other MPNs. ③ In the multivariate analysis, the complex karyotype（hazard ratio, 2.53; 95％ confidence interval, 1.06–6.05; *P*＝0.036）was independently associated with worse overall survival（OS）. Patients who received allogeneic stem cell transplantation（allo-HSCT）（median OS, 21.3 *vs*. 3 months; *P*＝0.05）or acute myeloid leukemia-like（AML-like）therapy（median OS, 13 *vs*. 3 months; *P*＝0.011）had significantly better OS than those who received supportive therapy.

**Conclusion:**

The proportions of patients with PMF-AP/BP with splenomegaly, myelofibrosis grade ≥2, a higher LDH level, and a shorter duration from diagnosis to the transformation to AP/BP were higher than those of patients with other Philadelphia-negative MPN-AP/BP. The complex karyotype was an independent prognostic factor for OS. Compared with supportive therapy, AML-like therapy and allo-HSCT could prolong the OS of patients with MPN-AP/BP.

费城染色体（Ph染色体）阴性骨髓增殖性肿瘤（MPN）[1]包括真性红细胞增多症（PV）、原发性血小板增多症（ET）、原发性骨髓纤维化（PMF）和骨髓增殖性肿瘤-不能分类（MPN-U）等。Ph染色体阴性MPN患者可能进展为疾病的终末阶段——骨髓增殖性肿瘤-加速期/急变期（MPN-AP/BP）。既往研究表明，MPN-AP/BP患者预后不良，总生存（OS）期较短[2]–[6]，异基因造血干细胞移植（allo-HSCT）是目前唯一的治愈方法，但移植相关并发症导致的高死亡率限制了其临床应用[7]。小样本量研究表明小剂量化疗或去甲基化药物（HMA）单药[8]及联合其他药物（芦可替尼、维奈克拉）[9]–[12]在MPN-AP/BP患者中具有一定疗效。本研究对我中心67例Ph染色体阴性MPN-AP/BP患者临床特征及预后因素进行回顾性分析。

## 病例与方法

一、病例

2014年2月至2021年12月期间于中国医学科学院血液病医院就诊的67例Ph染色体阴性MPN-AP/BP患者纳入本研究。纳入标准：①MPN-AP患者：既往MPN诊断符合世界卫生组织（WHO）2016版诊断分型标准[1]，患者骨髓或外周血原始细胞比例为10％～19％。②MPN-BP患者：既往MPN诊断符合WHO 2016版诊断分型标准[1]，患者骨髓或外周血原始细胞比例≥20％。③有完整的确诊时临床及实验室检查数据。

二、研究参数

患者进入AP/BP时性别、年龄等一般资料；症状、体征及红细胞/血小板输注情况等临床表现；血常规、骨髓穿刺涂片细胞形态学检查、骨髓活检组织切片病理学检查、染色体核型分析、二代测序基因突变检测等实验室检查资料。

三、染色体核型分析

短期培养法常规制备染色体标本，采用R显带法分析核型，并根据《人体细胞遗传学国际命名体制（ISCN2016）》进行核型描述。至少3个中期分裂相出现同一染色体缺失、至少2个中期分裂相出现同一染色体获得或同一结构异常判定为克隆性异常核型。

四、基因突变分析

取患者骨髓，分离单个核细胞，常规提取DNA并制备DNA全基因组文库。使用PCR引物扩增目的基因组，将目标区域DNA富集后，采用Illumina Hiseq测序平台进行测序。等位基因突变频率（VAF）≥2％的基因突变纳入分析，所有检测出的外显子区通过千人基因组计划、COSMIC（癌症中的体细胞突变目录）及PolyPhen-2数据库筛选出致病基因。具体方法见文献[13]。

五、预后评估

采用动态国际预后积分系统（DIPSS）[14]对PMF-AP/BP患者进行预后评估分析。DIPSS预后积分标准：年龄>65岁、WBC≥25×10^9^/L、有体质性症状［在确诊PMF前1年内体重下降10％和（或）不能解释的发热或重度盗汗持续超过1个月］、外周血原始细胞比例≥1％赋值1分，HGB<100 g/L赋值2分。低危组：0分；中危-1组：1～2分；中危-2组：3～4分；高危组：≥5分。

六、随访

所有病例随访至2022年4月15日，随访资料来源于住院/门诊病历及电话随访记录。OS期按患者进入AP/BP时间起至死亡或随访截止日期计算。

七、统计学处理

应用SPSS24.0软件包进行数据分析。偏态分布计量资料以“*M*（范围）”表示，组间比较采用非参数Mann-Whitney *U*检验。率的比较采用卡方检验或Fisher确切概率法。生存分析采用Kaplan-Meier法，单因素分析采用Log-rank检验，多因素分析采用Cox回归风险模型。以双侧*P*<0.05为差异有统计学意义。

## 结果

一、临床特征

67例Ph染色体阴性MPN-AP/BP患者中，男31例（46.3％），女36例（53.7％），中位年龄为60（33～75）岁；MPN-AP 26例（38.8％），MPN-BP 41例（61.2％）。进展为MPN-AP/BP时血常规（中位数）：HGB 82（36～208）g/L，WBC 14.58（0.58～182.51）×10^9^/L，PLT 81（2～892）×10^9^/L；乳酸脱氢酶（LDH）782.7（204.1～3150.1）U/L，中位外周血原始细胞比例15％（0～89％），中位骨髓原始细胞比例22.0％（2.5％～85.0％），输血依赖23例（34.3％）。5例患者既往已行脾切除术，其余62例患者中46例（74.2％）肋缘下可触及肿大脾脏。67例患者中60例有可供分析的染色体核型结果，其中染色体核型异常36例（60.0％）；36例染色体核型异常患者中13例（36.1％）为复杂染色体核型，最常见的5种染色体核型异常为：+8（19.4％，7例）、+1（16.7％，6例）、der（1:7）（11.1％，4例）、−7（8.3％，3例）、−17（8.3％，3例）。

67例MPN-AP/BP患者中，48例（71.7％）由PMF进展而来（PMF-AP/BP组），19例（28.3％）由其他MPN进展而来（其他MPN-AP/BP组），其中PV 6例（8.9％），ET 11例（16.4％），MPN-U进展2例（3％）。PMF-AP/BP组、其他MPN-AP/BP组LDH水平分别为925.9 U/L、576.2 U/L（*P*＝0.011），脾肿大患者占比分别为81.4％、57.9％（*P*＝0.05），骨髓网状纤维（MF）≥2级患者占比分别为93.6％、63.2％（*P*＝0.004），从诊断MPN至进展为AP/BP时间分别为28.7、81.0个月（*P*＝0.001）（表1）。

**表1:** PMF-AP/BP组与其他MPN-AP/BP组临床特征比较

临床特征	PMF-AP/BP（48例）	其他MPN-AP/BP（19例）	*P*值
男性［例（%）］	25（52.1）	6（31.6）	0.129
年龄［岁，*M*（范围）］	60（33~75）	59（47~75）	0.797
HGB［g/L，*M*（范围）］	82（36~122）	88（53~208）	0.440
WBC［×10^9^/L，*M*（范围）］	15.10（0.58~144.38）	13.90（1.55~182.51）	0.531
PLT［×10^9^/L，*M*（范围）］	83.5（2~892）	70（9~569）	0.370
白蛋白［g/L，*M*（范围）］	37.4（28.2~50.5）	39.9（29.3~46.8）	0.215
乳酸脱氢酶［U/L，*M*（范围）］	925.9（204.1~3150.1）	576.2（246.4~1260.0）	0.011
外周血原始细胞［%，*M*（范围）］	15（1~81）	14（0~89）	0.972
骨髓原始细胞［%，*M*（范围）]	22.0（2.5~69.0）	22.0（4.5~85.0）	0.784
脾脏肿大［例（%）］	35（81.4）	11（57.9）	0.050
体质性症状［例（%）］	27（56.3）	8（42.1）	0.296
输血依赖［例（%）］	18（37.5）	5（26.3）	0.385
进展为AP/BP时间［月，*M*（范围）］	28（1~150）	81（10~241）	0.001
复杂染色体核型［例（%）］	8（18.6）	5（29.4）	0.360
骨髓网状纤维≥2级［例（%）］	44（93.6）	12（63.2）	0.004

**注** PMF-AP/BP：原发性骨髓纤维化加速期/急变期；MPN-AP/BP：骨髓增殖性肿瘤（MPN）加速期/急变期

二、分子生物学特征

67例MPN-AP/BP患者的驱动基因突变中，JAK2V617F突变41例（61.2％），CALR外显子9突变13例（19.4％）（其中1型CALR突变11例，2型CALR突变2例），MPLW515突变3例（4.5％），未检测到JAK2、MPL和CALR基因突变10例（14.9％）。分别对驱动基因（JAK2V617F、CALR和MPL）VAF与外周血HGB、WBC、PLT、外周血原始细胞比例、骨髓原始细胞比例、血清白蛋白水平、LDH水平、是否有肋缘下可触及的脾脏肿大、是否有体质性症状、是否有复杂染色体核型、是否输血依赖进行相关性分析，结果表明仅JAK2V617F突变VAF与外周血WBC呈正相关（*r*＝0.441，*P*＝0.006），与其他临床特征无相关性；未发现CALR外显子9突变、MPLW515突变VAF与临床特征之间存在相关性。

64例MPN-AP/BP患者应用二代测序方法进行非驱动基因突变检测，基因突变图谱见图1。其中检出率较高的依次为ASXL1（42.2％，27例）、SRSF2（25％，16例）、SETBP1（22.6％，15例）、TET2（20.3％，13例）、RUNX1（20.3％，13例）、TP53（17.2％，11例）。在PMF-AP/BP组最常见的基因突变依次为ASXL1（51.1％，23例）、SRSF2（26.7％，12例）、TET2（22.2％，10例）、RUNX1（22.2％，10例）、SETBP1（22.2％，10例）；其他MPN-AP/BP组最常见的基因突变依次为SETBP1（26.3％，5例）、ASXL1（21.1％，4例）、SRSF2（21.1％，4例）、TP53（21.1％，4例）、RUNX1（15.8％，3例）。PMF-AP/BP组ASXL1基因突变检出率高于其他MPN-AP/BP组（51.1％对21.1％，*P*＝0.03）（图2），SRSF2基因突变中位VAF值高于其他MPN-AP/BP组［48.8％（44.8％～58.1％）对39.6％（7.9％～46.1％），*P*＝0.008］。

**图1:**
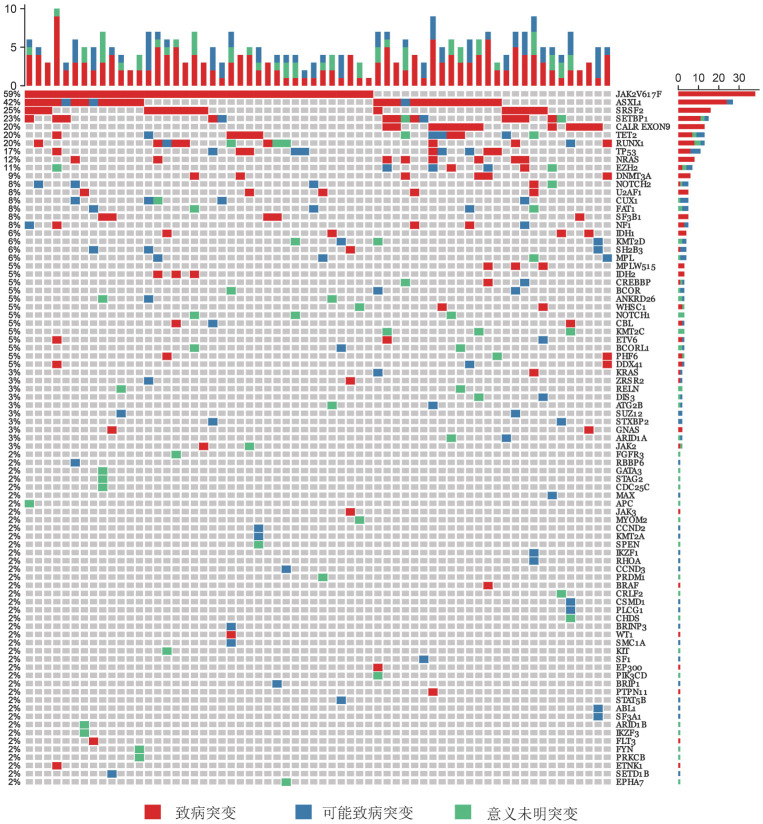
64例Ph染色体阴性骨髓增殖性肿瘤加速期/急变期（MPN-AP/BP）患者二代测序基因突变图谱

**图2:**
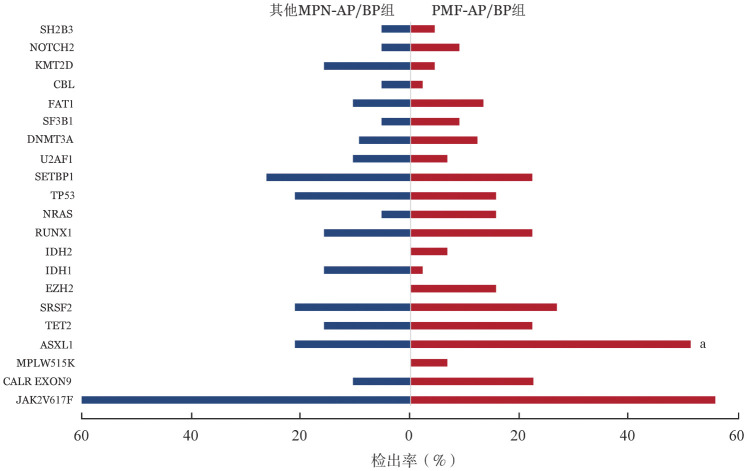
PMF-AP/BP组与其他MPN-AP/BP组基因突变检出率比较（^a^*P*<0.05） PMF-AP/BP：原发性骨髓纤维化加速期/急变期；MPN-AP/BP：骨髓增殖性肿瘤（MPN）加速期/急变期

67例患者中有15例可追溯到进展为AP/BP期前（慢性期）通过二代测序检测的驱动基因VAF和非驱动基因突变检测结果。对这15例患者进展到AP/BP期与慢性期驱动基因VAF进行比较分析，9例JAK2V617F突变的患者中，7例（77.7％）患者VAF在AP/BP时升高；4例CALR突变患者的VAF在AP/BP时均下降，2例MPLW515突变患者VAF在AP/BP前后无明显变化。与慢性期相比，15例患者中9例（60.0％）在AP/BP时获得1种以上新的非驱动基因突变，检出比例较高的突变基因依次为RUNX1（26.6％，4例）、ASXL1（13.3％，2例）、NRAS（13.3％，2例）、ETV6（13.3％，2例）。

三、MPN-AP/BP患者预后影响因素分析

至随访截止，67例MPN-AP/BP患者中17例存活，44例死亡，6例失访，共61例MPN-AP/BP患者纳入生存分析，中位OS期为11.00（95％*CI* 8.02～13.97）个月。单因素分析结果显示，影响MPN-AP/BP患者OS的不良预后因素有复杂染色体核型（*HR*＝4.40，95％*CI* 1.97～9.80，*P*<0.001）、MF ≥2级（*HR*＝4.09，95％*CI* 1.24～13.49，*P*＝0.011）、可触及的脾脏肿大（*HR*＝2.77，95％*CI* 1.16～6.60，*P*＝0.015）、HGB<90 g/L（*HR*＝2.38，95％*CI* 1.14～4.96，*P*＝0.015）、PLT≤350×10^9^/L（*HR*＝3.21，95％*CI* 1.24～8.35，*P*＝0.01）。将单因素分析*P*<0.05的因素纳入COX多因素分析，结果显示复杂染色体核型（*HR*＝2.53，95％*CI* 1.06～6.05，*P*＝0.036）是影响MPN-AP/BP患者OS的独立不良预后因素。详见表2。

**表2:** 61例骨髓增殖性肿瘤加速期/急变期（MPN-AP/BP）患者预后影响因素分析

临床特征	单因素分析			多因素分析		
	*HR*	95%*CI*	*P*值	*HR*	95%*CI*	*P*值
男性	1.40	0.77~2.54	0.256			
年龄>65岁	1.33	0.66~2.68	0.417			
HGB<90 g/L	2.38	1.14~4.96	0.015	1.55	0.64~3.78	0.331
WBC≥25×10^9^/L	1.07	0.54~2.08	0.850			
PLT≤350×10^9^/L	3.21	1.24~8.35	0.010	1.98	0.74~5.30	0.172
白蛋白<35 g/L	1.39	0.74~2.60	0.292			
诊断为原发性骨髓纤维化	1.79	0.86~3.74	0.106			
脾脏肿大	2.77	1.16~6.60	0.015	1.65	0.57~4.79	0.355
输血依赖	1.25	0.67~2.32	0.473			
复杂染色体核型	4.40	1.97~9.80	<0.001	2.53	1.06~6.05	0.036
外周血原始细胞比例≥10％	1.23	0.61~2.51	0.544			
骨髓原始细胞比例≥10％	2.65	0.92~7.62	0.055			
有体质性症状	1.05	0.58~1.91	0.858			
骨髓网状纤维≥2级	4.09	1.24~13.49	0.011	2.48	0.55~11.16	0.237
动态国际预后积分系统						
中危-2组	0.84	0.33~2.12	0.705			
高危组	1.21	0.40~3.63	0.737			
中危-1组（对照）						
JAK2V617F突变	0.92	0.49~1.72	0.788			
1型CALR突变	0.65	0.29~1.49	0.300			
驱动基因突变阴性	1.32	0.55~3.17	0.519			
TP53突变	2.18	0.94~5.04	0.057			
ASXL1突变	0.88	0.47~1.67	0.697			
SRSF2突变	1.18	0.58~2.38	0.646			
SETBP1突变	0.97	0.46~2.04	0.928			
RUNX1突变	0.84	0.39~1.84	0.659			
TET2突变	1.56	0.76~3.22	0.214			
EZH2突变	0.51	0.16~1.68	0.254			

58例患者有进展为AP/BP后治疗情况随访结果。其中12例患者接受支持治疗[6]，包括输血、口服降细胞和缩脾药物（美法仑、羟基脲和芦可替尼）；42例患者接受包括IA（去甲氧柔红霉素+阿糖胞苷）/DA（柔红霉素+阿糖胞苷）方案化疗和去甲基化药物（HMA）等白血病样化疗[6]；4例患者接受allo-HSCT。支持治疗组的中位OS期为3.0（95％ *CI* 2.3～3.7）个月，白血病样化疗组的中位OS期为13.0（95％ *CI* 8.3～17.7）个月，差异有统计学意义（*P*＝0.011）。allo-HSCT组的中位OS期为21.3（95％ *CI* 10.2～32.3）个月，优于支持治疗组（*P*＝0.05），与白血病样化疗组比较差异无统计学意义（*P*＝0.357）。为进一步分析MPN-AP/BP患者中影响预后的临床特征（复杂染色体核型）与不同治疗方案对预后的相互作用，采用Cox回归风险模型进一步分析，结果显示复杂染色体核型仍是影响MPN-AP/BP患者OS的独立不良预后因素（*HR*＝4.58，95％ *CI* 1.96～10.70，*P*<0.001）；allo-HSCT和白血病样化疗（*HR*＝0.25，95％ *CI* 0.11～0.57，*P*＝0.001）仍是影响MPN-AP/BP患者OS的独立良好预后因素。

## 讨论

Ph染色体阴性MPN是一组起源于造血干细胞的恶性克隆性疾病，其特征为一系或多系成熟细胞过度增殖伴有向急性髓系白血病转化风险增高，是严重影响患者长期生存的主要危险因素之一[1]。既往国外多个回顾性研究表明，MPN-AP患者中位OS期短于2年，MPN-BP患者中位OS期短于6个月[15]。既往关于MPN-AP/BP患者的研究[2]–[5],[16]–[18]多为国外的小样本临床报道，为阐释我国MPN-AP/BP患者的临床特征及预后，我们总结分析了在我中心诊治的67例MPN-AP/BP患者的临床和分子生物学特征及其预后影响因素，以期进一步指导临床实践。

既往国外研究表明，由不同亚型MPN进展的MPN-AP/BP患者在临床特征上有差异[6]。本研究结果显示PMF-AP/BP较其他MPN-AP/BP患者LDH水平更高，脾肿大、MF≥2级患者占比更高；表明MPN患者进展到AP/BP期后，临床特征仍与其慢性期的疾病类型密切相关。ASXL1突变是PMF患者最常见非驱动基因突变，其检出率为21.7％～38.9％[19]–[20]，高于PV（12％）和ET（11％）患者[21]。本研究中PMF-AP/BP患者ASXL1突变检出率亦显著高于其他MPN-AP/BP患者，与Venton等[18]研究结果一致，可能与ASXL1突变在慢性期PMF患者中检出率较高有关。我们既往研究[13]表明，与PMF相比，PV/ET后骨髓纤维化患者中极少出现剪接体复合物相关基因突变（如U2AF1、SRSF2等）。本研究中PMF-AP/BP组SRSF2基因突变中位VAF值高于其他MPN-AP/BP组，提示作为亚克隆的SRSF2突变可能在其他MPN-AP/BP患者中获得时间晚于PMF-AP/BP患者，但目前尚不明确是慢性期存在的SRSF2突变还是AP/BP期新增的SRSF2突变最终促进了疾病进展。SRSF2突变获得时机对不同亚型MPN患者进展为AP/BP的影响仍需在大样本量序贯患者中进一步研究。

国外有研究表明，接受芦可替尼治疗的PMF患者中，JAK2V617F突变VAF增高或出现高分子风险突变（HMR）是导致芦可替尼缩脾疗效丧失比例增高的因素之一；出现克隆进展的患者中位生存时间明显缩短[22]。同样有研究表明，非驱动基因克隆演变是促使慢性期MPN向急变期转化的重要因素，其中ASXL1、EZH2、TP53等是常见的新增非驱动基因突变[16]–[18]。在本研究中，仅15例患者可序贯比较慢性期和AP/BP期驱动基因VAF和非驱动基因突变结果，结果显示疾病转化过程中伴有JAK2V617F突变VAF增高和新增非驱动基因突变。上述研究结果亦表明在MPN慢性期进行驱动基因检测和非驱动基因突变检测的重要性。我们既往的研究[20]表明，包含HMR突变的MIPSS70-plus预后积分系统对PMF患者预后评估的价值优于DIPSS。因此建议慢性期MPN患者应定期行二代测序基因突变检测，既可作出更准确的预后评估，又可及时预判疾病进展的风险。

国外的回顾性研究结果显示，MPN-AP/BP患者独立的不良预后因素包括PLT<100×10^9^/L、输血依赖、年龄>65岁、低白蛋白水平、基因突变数目≥4个、复杂染色体核型和TP53、RUNX1、PTPN11、SRSF2、TET2基因突变等[5]–[6],[16]–[18]。本研究中我们仅证实复杂染色体核型为MPN-AP/BP患者独立不良预后因素，可能与本研究样本量较小有关。期望通过开展多中心大样本量研究建立适用于我个MPN-AP/BP患者的预后积分系统。

国外的回顾性研究表明，不同治疗方案亦是影响MPN-AP/BP患者的生存因素之一，其中接受支持治疗的患者中位生存期仅为1.1～3个月[3]–[6]，接受白血病样化疗的患者中位OS期为9～11.8个月[4]–[5],[23]–[24]；接受allo-HSCT和白血病样化疗的患者OS期无明显差异[5]–[6],[17]，表明allo-HSCT较白血病样化疗并不能显著改善MPN-AP/BP患者长期生存。这可能与接受allo-HSCT的MPN-AP/BP患者移植前诱导化疗难以达到完全缓解以及移植后高复发率相关[15]。我们的研究亦表明不同的治疗方案是影响MPN-AP/BP患者的独立预后因素，接受allo-HSCT或白血病样化疗的患者OS期差异无统计学意义，但均优于接受支持治疗患者。因此，不推荐MPN患者进入AP/BP后再行allo-HSCT治疗，而应在有进展为AP/BP高危因素时尽早行allo-HSCT治疗；对于MPN-AP/BP患者应尽可能接受白血病样化疗。

本研究结果初步揭示了我国Ph染色体阴性MPN-AP/BP患者的临床特征及预后因素，PMF-AP/BP患者与其他MPN-AP/BP患者比较，具有较高的脾大和MF≥2级发生率，LDH水平较高，进展为AP/BP较快；复杂染色体核型是MPN-AP/BP患者独立不良预后因素，而白血病样化疗和allo-HSCT较支持治疗可延长MPN-AP/BP患者OS期。本研究为单中心回顾性研究，纳入病例数量较少，上述结论尚需多中心、大样本量的研究进一步验证。
